# Supplementary material for: Committed sea-level rise under the Paris Agreement and the legacy of delayed mitigation action
Source: Nat Commun. 2018 Feb 20;9:601. doi: 10.1038/s41467-018-02985-8 (PMC5820313; doi:10.1038/s41467-018-02985-8)
Supplement: Supplementary file 1 — Supplementary Information [file 41467_2018_2985_MOESM1_ESM.pdf]

SUPPLEMENTARY INFORMATION

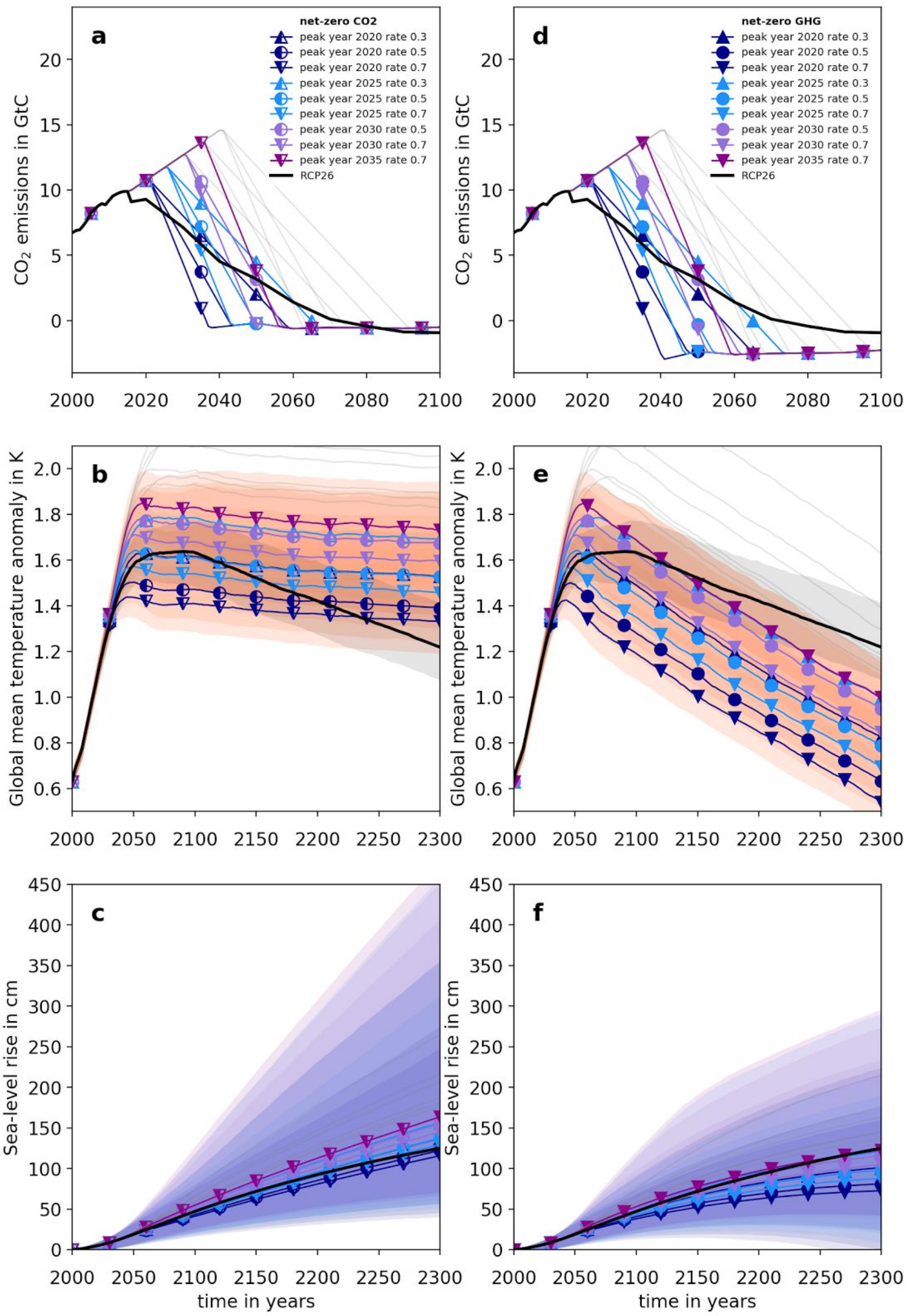

Supplementary Figure 1 | same as Fig. 1, but with RCP2.6 scenario overlaid.
Black line indicates RCP2.6 scenario. Colored lines: emission scenarios based on RCP2.6 with CO_2 emissions linearly continued with the present day rate until peak year. CO_2 emissions decline by 0.3, 0.5 and 0.7 GtC yr^{-2} thereafter until zero CO_2 (panel a) or net-zero greenhouse gas emissions (panel d) are reached. Scenarios that do not hold warming to below 2°C with at least 66% chance are masked grey. b,e: Global mean temperature responses to emissions scenarios in (a,d) in °C above pre-industrial. c,f: Global mean sea-level rise with respect to the year 2000. Shading refers to the central 66th percentile range per scenario in panels b,e and to the central 90th percentile range in panels c,f.

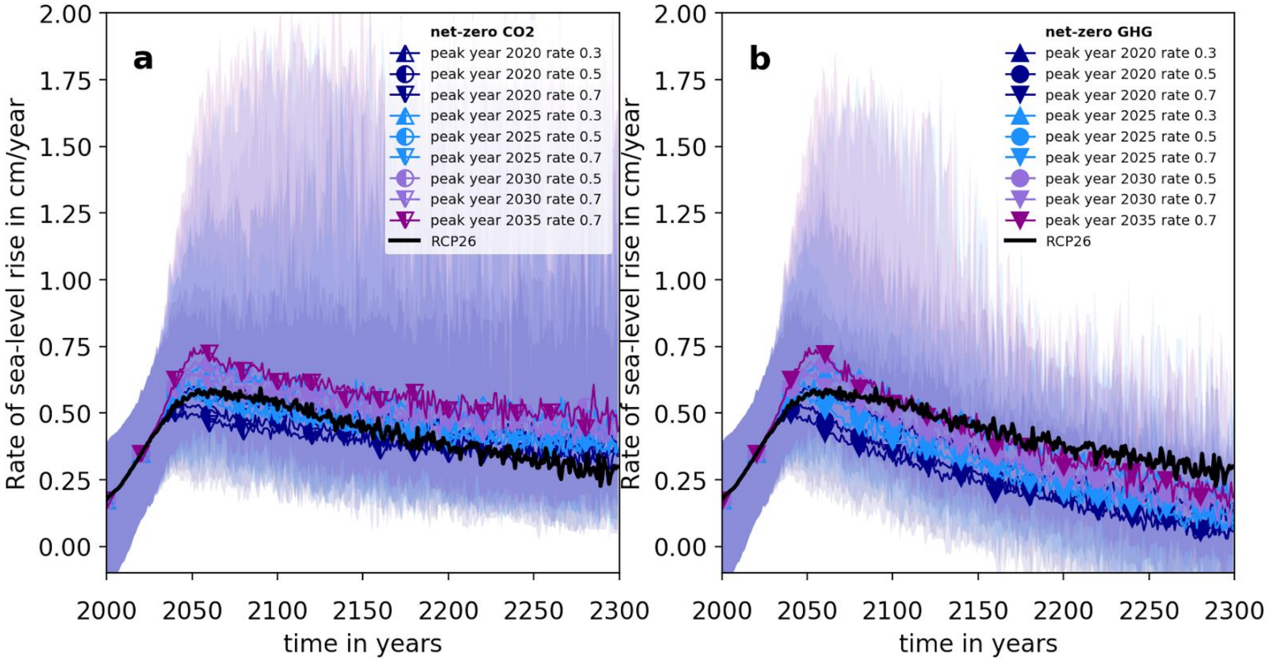

Supplementary Figure 2 | Rates of sea-level rise corresponding to Fig. 1 panels (c,f)
Rates of sea-level rise for (a) zero CO2 scenarios and (b) zero GHG scenarios. The RCP2.6 scenario is overlaid in black.

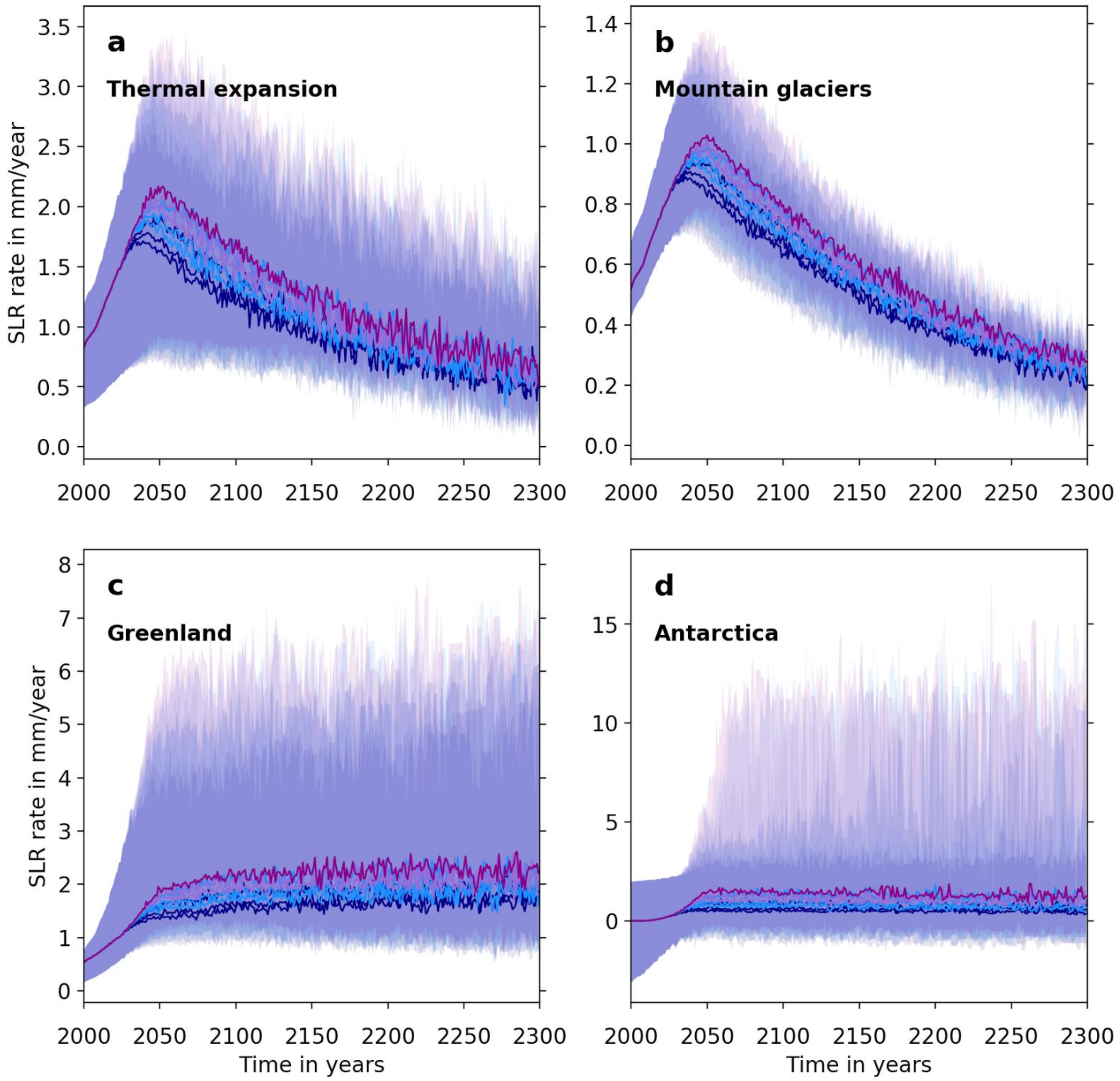

Supplementary Figure 3 | Rates of sea-level rise, corresponding to Fig. 2, net-zero CO_2 scenarios

Time series of the rates of the sea-level responses of thermal expansion (a), mountain glaciers (b), Greenland solid ice discharge (c), Greenland surface mass balance (d), Antarctic solid ice discharge (e) and Antarctic surface mass balance (f). Sea-level rise rates are in mm/year. Colors refer to peak years as in Fig. 1. Shadings show the central 90th percentile range.

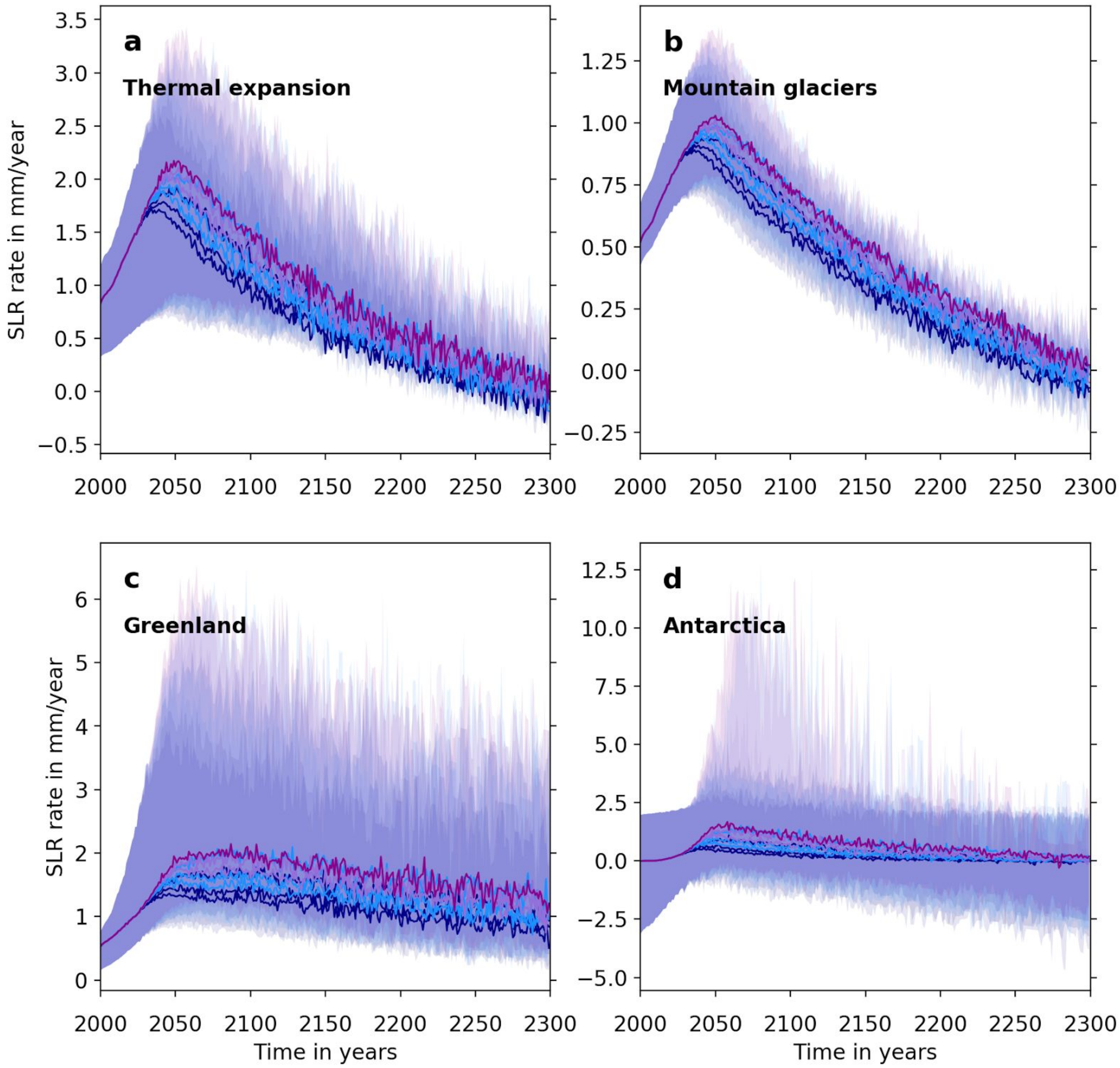

Supplementary Figure 4 | Rates of sea-level rise, corresponding to Fig. 3, net-zero GHG scenarios.

Time series of the rates of the sea-level responses of thermal expansion (a), mountain glaciers (b), Greenland solid ice discharge (c), Greenland surface mass balance (d), Antarctic solid ice discharge (e) and Antarctic surface mass balance (f). Sea-level rise rates are in mm/year. Colors refer to peak years as in Fig. 1. Shadings show the central 90th percentile range.
