# Supplementary material for: Committed sea-level rise under the Paris Agreement and the legacy of delayed mitigation action
Source: Nat Commun. 2018 Feb 20;9:601. doi: 10.1038/s41467-018-02985-8 (PMC5820313; doi:10.1038/s41467-018-02985-8)
Supplement: Supplementary file 3 — Description of Additional Supplementary Files [file 41467_2018_2985_MOESM3_ESM.docx]

**Description of Additional Supplementary Files**

File Name: Supplementary Data 1

Description: Fossil CO 2 emissions, total GHG emissions in GtC CO 2 equivalent and total GHG emissions in GtCO 2 equivalent for the year 2030.

File Name: Supplementary Data 2

Description: Sea-level rise in 2300 for scenarios consistent with current NDCs (total GHG emissions above 49 GtCO 2 eq/yr) relative to 2000 in cm. This is a subset of Supplementary Data 1.

File Name: Supplementary Data 3

Description: Sea-level rise per component for the RCP2.6 scenario in year 2300 relative to 2000 in cm.

File Name: Supplementary Data 4

Description: Sea-level rise per component in year 2300 relative to 2000 in cm. for net-zero CO2 scenarios

File Name: Supplementary Data 5

Description: Calibrated parameters for Greenland surface mass balance. Observations refer to (Broeke et al. 2016; Forsberg, Soerensen, and Simonsen 2017; Box and Colgan 2013).
